# Pyridine vs. Imidazole Axial Ligation on Cobaloxime Grafted Graphene: Hydrogen Evolution Reaction Insights

**DOI:** 10.3390/nano12173077

**Published:** 2022-09-05

**Authors:** Ioanna K. Sideri, Georgios Charalambidis, Athanassios G. Coutsolelos, Raul Arenal, Nikos Tagmatarchis

**Affiliations:** 1 Theoretical and Physical Chemistry Institute, National Hellenic Research Foundation, 116 35 Athens, Greece; 2 Chemistry Department, Laboratory of BioInorganic Chemistry, University of Crete, 710 03 Heraklion, Greece; 3 Laboratorio de Microscopias Avanzadas (LMA), Universidad de Zaragoza, 50018 Zaragoza, Spain; 4 Instituto de Nanociencia y Materiales de Aragon (INMA), CSIC-U. de Zaragoza, 50009 Zaragoza, Spain; 5 ARAID Foundation, 50018 Zaragoza, Spain

**Keywords:** cobaloxime, axial ligation, graphene, electrocatalysis, hydrogen evolution reaction

## Abstract

While cobaloximes have been protagonists in the molecular (photo)catalytic hydrogen evolution reaction field, researchers originally shed light on the catalytically active metallic center. However, the specific chemical environment of cobalt, including equatorial and axial ligation, has also a strong impact on the catalytic reaction. In this article, we aim to demonstrate how pyridine vs. imidazole axial ligation of a cobaloxime complex covalently grafted on graphene affects the hydrogen evolution reaction performance in realistic acidic conditions. While pyridine axial ligation mirrors a drastically superior electrocatalytic performance, imidazole exhibits a remarkable long-term stability.

## 1. Introduction

Mimicking nature has always been an attractive alternative to tackle unresolved scientific challenges. Inspired by vitamin B12 and in a quest to imitate the activity of hydrogenase enzymes (metalloproteins, which bidirectionally catalyze the interconversion between H_2_ and a pair of protons and electrons) [1], cobaloximes were conceptualized [2]. Akin to hydrogenase, their intrinsic extraordinary characteristics derive from their redox-active metal center (Co) and the closely lying polar oximes, coordinated in a square planar geometry. Compared to hydrogenase, a suitable electron supplement was the only missing piece, which enabled them to dominate in the molecular electrocatalytic H_2_ production research field [2,3]. However, certain limitations, such as their insufficient aqueous solubility and long-term instability, impede their usage in realistic conditions, i.e., aqueous media.

Artero and coworkers were the first to overcome this setback and perform an electrocatalytic H_2_ evolution reaction (HER) by grafting cobaloxime on the surface of a carbon nanotube (CNT) electrode, ensuring in this way high stability and oxygen tolerance [4,5,6]. Yet, due to the electrochemical fabrication of cobaloxime on CNTs already deposited on a gas-diffusion layer (GDL) electrode, the analytical and spectroscopic techniques available for the complete characterization of the final electrocatalyst were limited. Therefore, useful insights on the structural aspect, on the cobaloxime loading and on the true active species in HER, were suppressed albeit important. Indeed, it was demonstrated that the chemical structure and specifically the outer coordination sphere of the cobaloxime catalytic center plays an important role in the electrocatalytic H_2_ evolution performance of a polymer/cobaloxime-modified CNT electrocatalyst, that mimics the function of a protein matrix [7]. Earlier, the effect of differently-substituted pyridinic cobalt axial ligands to the H_2_ evolution catalytic activity of the complex was also studied, on a molecular level [8]. Interestingly, electron donating pyridine ligands result in enhanced catalytic currents. The influence of the cobalt axial ligand is also significant in photochemical hydrogen production [9], where the performance is linked to the strength of the Co-N ligation; weaker axial ligation results in rapid initial hydrogen evolution catalysis but low stability. On the contrary, stronger axial ligation induces a lower initial rate, but an elevated long-term stability.

Of course, the studies on a molecular level refer to homogeneous catalytic hydrogen production, performed in either non-aqueous conditions or neutral pH aqueous buffer. But how about the influence of the cobaloxime axial ligation on the electrocatalytic HER performance in a realistic set-up? Herein, we aspire to take this research field one step further by attempting to answer this query. To do so, we envisioned a rationally designed heterogeneous electrocatalytic system, compatible with practical aqueous acidic conditions, in order to study the HER performance of two twin electrocatalysts with the sole difference being their axial ligation to the cobaloxime complex. Covalent functionalization of graphene was selected as an ideal route to ensure the required stability for cobaloxime under such conditions, while pyridine and imidazole functionalities were selected as the axial ligands, based on their versatility, occurrence and different electron-donating ability. Indeed, the two different conjugate π systems that shape the outer coordination sphere of Co alter the HER performance of the catalyst, resulting in an overall elevated performance for pyridine axial ligation, but in better stability and endurance for imidazole axial ligation.

## 2. Materials and Methods

### 2.1. Instrumentation

*^1^H and ^13^C NMR spectra* were acquired on a Varian 600 or 300 MHz (150.9 or 75.5 MHz, respectively, for ^13^C) NMR spectrometer at ambient temperature and were internally referenced to residual solvent signals. Data for ^1^H NMR are reported as follows: chemical shift (*δ* ppm), multiplicity (s = singlet, br s = broad singlet, d = doublet, dt = doublet of triplets, dd = doublet of doublet, t = triplet, m = multiplet), coupling constant and integration. Data for ^13^C NMR are reported in terms of chemical shift (*δ* ppm).

*Tip sonication* was performed with a Bandelin Sonoplus Ultrasonic Homogenizer HD 3200 equipped with a flat head probe (VS70T), running at 35% of the maximum power (250 W). *Microwave synthesis* was performed with a CEM Discover microwave reactor equipped with infrared pyrometer and pressure control system. During the synthesis, the microwave system was operated in a dynamic mode, where the power was automatically adjusted to maintain the set temperature.

*Mid-infrared spectra* in the region 500–4500 cm^−1^ were acquired on a Fourier transform IR spectrometer (Equinox 55 from Bruker Optics) equipped with a single reflection diamond ATR accessory (DuraSamp1IR II by SensIR Technologies). Typically, 100 scans were acquired at 2 cm^−1^ resolution.

*Micro-Raman spectroscopy scattering* measurements were performed at room temperature in the backscattering geometry using a RENISHAW in Via Raman spectrometer, equipped with a CCD camera and a Leica microscope. A 1200 lines mm^−1^ grating was used for all measurements, providing a spectral resolution of ±1 cm^−1^. As an excitation source, the Ar^+^ laser 514 nm with less than 2.65 mW laser power was used. Measurements were taken with 10 s of exposure times at varying numbers of accumulations. The laser spot was focused on the sample surface using a long working distance 50× objective. Raman spectra were collected after map image acquisition was conducted on various (3–5) areas of the sample and recorded with Peltier cooled CCD camera. The intensity ratio I_D_/I_G_ was obtained by taking the peak intensities following any baseline corrections. For the mapping recordings, 5–10 areas of 121 acquisition points each were scanned for every sample and we present here a representative one close to the total average with respect to the intensity ratio I_D_/I_G_. The data were collected and analyzed with Renishaw Wire and Origin software.

*Thermogravimetric analysis* was performed using a TGA Q500 V20.2 Build 27 instrument by TA in an inert atmosphere of nitrogen (purity > 99.999%). In a typical experiment, 2 mg of the material was placed in the platinum pan and the temperature was equilibrated at 40 °C. Subsequently, the temperature was increased to 900 °C with a rate of 10 °C/min and the mass changes were recorded as a function of temperature.

*UV-Vis absorption spectra* were recorded on a PerkinElmer (Lambda 19) UV-Vis-NIR spectrophotometer.

*High-resolution scanning transmission electron microscopy (HRSTEM)* and *electron energy loss spectroscopy (EELS)* analyses were performed in a probe-corrected FEI Titan-Low-Base 60−300 operating at 80 kV (equipped with a X-FEG^®^ gun and Cs-probe corrector (CESCOR from CEOS GmbH)). EELS studies were performed using the spectrum-image/-line modes. The powders were dispersed in ethanol and the suspensions were ultrasonicated and dropped onto copper carbon holey grids.

*X-ray photoelectron spectroscopy (XPS)* measurements were acquired using a Kratos Axis Supra spectrometer equipped with a monochromated Al Kα X-ray source using an analyzer pass energy of 160 eV for survey spectra and 20 eV for the core level spectra. Spectra were recorded by setting the instrument to the hybrid lens mode and the slot mode providing approximately a 700 × 300 µm^2^ analysis area using charge neutralization. Regions were calibrated using the reference value BE (C1s sp^2^) = 284.5 eV. All XPS spectra were analyzed using CASA XPS software. The XPS peaks were fitted to GL(70) Voigt lineshape (a combination of 70% Gaussian and 30% Lorentzian character), after performing a Shirley background subtraction.

*Hydrogen evolution reaction (HER) measurements* were carried out using an Autolab PGSTAT128N potentiostat/galvanostat and were carried out at room temperature in a standard three-compartment electrochemical cell by using a graphite rod as a counter-electrode, an RDE with glassy carbon disk (geometric surface area: 0.196 cm^2^) as a working electrode, and Hg/HgSO_4_ (0.5 M K_2_SO_4_) as reference electrode. LSV measurements for HER were carried out at room temperature in N_2_-saturated aqueous 0.5 M H_2_SO_4_. The catalyst ink was prepared by dispersing 4.0 mg of the catalytic powder in an 1 mL mixture of deionized water, isopropanol, and 5% Nafion (*v*/*v*/*v* = 4:1:0.02) and sonicated for 30 min prior use. Before casting the electrocatalytic ink on the electrode’s surface, the working electrode was polished with 6, 3, and 1 μm diamond pastes, rinsed with deionized water, and sonicated in double-distilled water. Afterward, 8.5 µL aliquots of the electrocatalyst were casted on the electrode surface and were left to dry at room temperature. EIS measurements were conducted from 10^5^ to 10^−1^ Hz with an AC amplitude of 0.01 V. The EIS measurements (Nyquist plots) were recorded at the low overpotential (kinetic) region.

*Cyclic*, *differential pulse* and *squarewave voltammograms* were recorded on an Autolab PGSTAT128 N potentiostat/galvanostat equipped with a dual mode bipotentiostat (BA module) electrochemical analyzer using a three-electrode system. A platinum button electrode was used as the working electrode. A platinum cloth served as the counter electrode and a platinum wire was used as the reference electrode. Ferrocene/ferrocenium redox couple was used as an internal standard. All solutions were purged prior to electrochemical and spectral measurements using nitrogen gas.

### 2.2. Experimental

All solvents and reagents were purchased from Sigma-Aldrich (St. Louis, MO, USA) and used without further purification unless stated otherwise. Anhydrous dichloromethane was prepared by distillation over CaH_2_. ^1^H and ^13^ C-NMR spectra of all synthesized (and previously unreported) compounds are provided as Supplementary Material (Figures S1–S10).

#### 2.2.1. Synthetic Procedures

##### (4-(Pyridin-4-yl-carbamoyl)phenyl)carbamate (**2**)

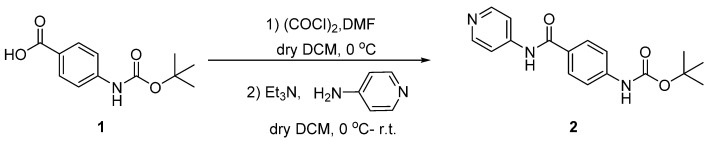



To a stirring suspension of 4-((*tert*-butoxycarbonyl)amino)benzoic acid (**1**) [10] (0.71 g, 3.0 mmol) in 10 mL of freshly distilled anhydrous dichloromethane (DCM) were added oxalyl chloride (0.62 mL, 6 mmol) and *N*,*N*-dimethylformamife (DMF) (2 drops) at 0 °C. The reaction mixture was stirred for 4 h at 0 °C under N_2_. The solvent and the excess of oxalyl chloride were removed in vacuo to afford the intermediate acid chloride as a light yellow solid (0.75 g, 3 mmol, 98%) [11]. The solid residue was then dissolved in 10 mL of freshly distilled anhydrous DCM, cooled to 0 °C, and dropwise added to a stirring dispersion of 4-aminopyridine (0.31 g, 3.3 mmol) and triethylamine (0.5 mL, 3.6 mmol) in 10 mL of freshly distilled anhydrous DCM at 0 °C. The color of the solution turned immediately bright yellow upon acid chloride addition and HCl(g) release was observed. The reaction mixture was allowed to warm to room temperature (r. t.) slowly and stirred overnight under N_2_. The progress of the reaction was monitored by thin layer chromatography (TLC) analysis in ethyl acetate (EtOAc). After completion upon TLC analysis, the reaction was quenched with brine (10 mL) and the aqueous layer was extracted with ethyl acetate (3 × 5 mL), chloroform (2 × 5 mL), dried over sodium sulfate, filtered and concentrated in vacuo. Compound **2** was purified by column chromatography in EtOAc and isolated as an off-white solid (0.49 g, 1.6 mmol, 53% over two steps).  ^1^H NMR (300 MHz, DMSO-*d*_6_) *δ* 10.42 (s, 1 H), 9.75 (s, 1 H), 8.45 (d, *J* = 5.2 Hz, 2 H), 7.91 (d, *J* = 8.7 Hz, 2 H), 7.77 (d, *J* = 5.9 Hz, 2 H), 7.61 (d, *J* = 8.6 Hz, 2 H), 1.49 (s, 9 H); ^13^C (75.5 MHz, DMSO-*d*_6_) *δ* 166.19, 152.96, 150.65, 146.49, 143.63, 129.31, 127.6, 117.53, 114.34, 80.07 and 28.46.

##### 4-Amino-*N*-(pyridin-4-yl)benzamide (**3**)

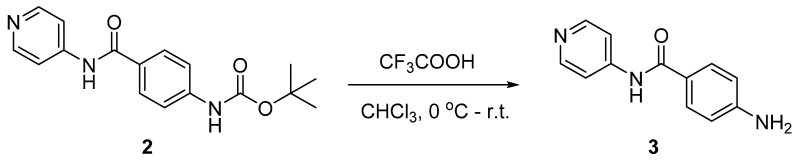



To a stirring suspension of **2** (0.49 g, 1.6 mmol) in chloroform (14.5 mL) in 0 °C, trifluoroacetic acid (3.6 g, 31.4 mmol) was dropwise added. The reaction was allowed to warm to r.t. and was left stirring overnight. The progress of the reaction was monitored by TLC (90/10 CHCl_3_/MeOH). Upon completion, the reaction was quenched with H_2_O and saturated aqueous solution of Na_2_CO_3_ was then added until pH~8. A white precipitate was formed and the aqueous layer was thoroughly extracted with CHCl_3_ (3 × 10 mL) and EtOAc (3 × 10 mL) dried over sodium sulfate, filtered and concentrated in vacuo. Compound **3** was isolated as a white solid without further purification (0.3 g, 1.4 mmol, 89%). ^1^H NMR (300 MHz, DMSO-*d*_6_) *δ* 10.10 (s, 1 H), 8.41 (d, *J* = 5.1 Hz, 2 H), 7.75 (m, 4 H), 6.61 (d, *J* = 8.5 Hz, 2 H), 5.90 (s, 2 H); ^13^C (75.5 MHz, DMSO-*d*_6_) *δ* 166.04, 152.77, 150.12, 146.57, 129.77, 120.10, 113.76 and 112.56.

##### *N*-(3-(1H-Imidazol-1-yl)propyl)-4-aminobenzamide (**5**)

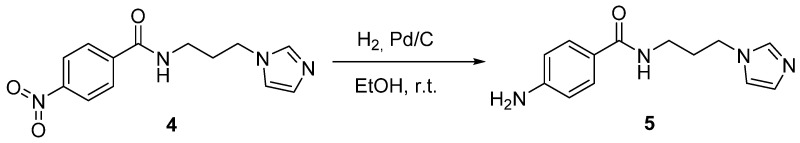



To a stirring solution of *N*-(3-(1H-imidazol-1-yl)propyl)-4-nitrobenzamide (**4**) [12] (0.45 g, 1.6 mmol) in ethanol (30 mL), 10% Pd/C (0.045 g) catalyst was added. The flask was carefully degassed in vacuo and then N_2_ flushed. This procedure was repeated three times. The flask was then evacuated, flushed with H_2_, put under balloon pressure of H_2_ and left stirring for 3 h. The progress of the reaction was monitored by TLC (90/10 CHCl_3_/MeOH). Upon completion, the reaction mixture was filtered through celite to remove the catalyst and the residue was washed with ethanol. The filtrate was then concentrated in vacuo to yield compound **5** as an off-white solid (0.39 g, 1.6 mmol, 100%). ^1^H NMR (600 MHz, CD_3_OD-*d*_4_) *δ* 7.71 (s, 1 H), 7.59 (d, *J* = 8.6 Hz, 2 H), 7.18 (s, 1 H), 6.97 (s, 1 H), 6.66 (d, *J* = 8.6 Hz, 2 H), 4.10 (t, *J* = 7.0 Hz, 2 H), 3.35 (t, *J* = 6.7 Hz, 2 H), 2.11–2.01 (m, 2 H); ^13^C (150.9 MHz, CD_3_OD-*d*_4_) *δ* 170.63, 153.26, 138.49, 129.91, 129.01, 123.02, 120.65, 114.67, 45.77, 37.94 and 32.29.

##### Synthesis of Cobaloxime Complexes (**6**) and (**7**) [13]

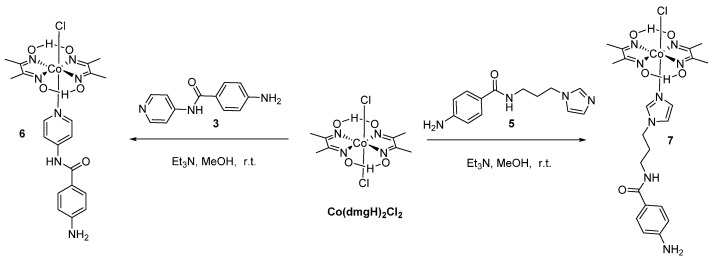



The general synthesis of cobaloxime complexes, is adapted from previously reported procedures [13]. Briefly, to a stirring suspension of Co(dmgH)_2_Cl_2_ [13] (0.2 g, 0.55 mmol) in MeOH (35 mL), Et_3_N (0.056 g, 0.55 mmol) was added. The dark green suspension immediately turned brown upon Et_3_N addition and was left stirring for 15 min in r.t. The pyridine or imidazole adduct was then added (0.46 mmol) dispersed in MeOH (15 mL). The solution was left stirring at r.t. for 3 h. In the case of pyridine adduct, solid precipitated after 15 min and upon completion of the 3 h period, complex **6** (pyridine/Co(III)) was collected by filtration, washed with ice cold MeOH (1 mL) and dried. Cobaloxime adduct **6** was collected as a brown solid (0.21 g, 0.39 mmol, 89%). ^1^H NMR (600 MHz, DMSO-*d*_6_) *δ* 18.43 (s, 2 H), 10.41 (s, 1 H), 7.77 (d, *J* = 5.8 Hz, 2H), 7.71 (d, *J* = 5.8 Hz, 2H), 7.66 (d, *J* = 8.1 Hz, 2H), 6.57 (d, *J* = 8.2 Hz, 2H), 5.98 (s, 2 H), 2.32 (s, 12 H); ^13^C (150.9 MHz, DMSO-*d*_6_) *δ* 166.43, 153.52, 152.70, 150.12, 149.52, 130.34, 119.23, 115.79, 112.82 and 12.85. In the case of imidazole adduct, after the 3 h period, the reaction mixture was concentrated in vacuo to yield a brown oil. Repeated recrystallization from MeOH afforded pure compound **7** (imidazole/Co(III)) as a brown solid (0.13 mg, 0.24 mmol, 52%). ^1^H NMR (300 MHz, DMSO-*d*_6_) *δ* 18.63 (s, 2 H), 7.96 (s, 1 H), 7.53 (d, *J* = 7.6 Hz, 2 H), 7.38 (s, 1 H), 7.21 (s, 1 H), 6.53 (d, *J* = 7.4 Hz, 2 H), 6.42 (s, 1 H), 5.61 (s, 2 H), 3.97 (s, 2 H), 2.97 (s, 2 H), 2.32 (s, 12 H), 1.74 (s, 2 H); ^13^C (75.5 MHz, DMSO-*d*_6_) *δ* 166.37, 151.62, 151.45, 137.55, 128.69, 126.66, 121.96, 112.47, 45.53, 35.67, 30.49 and 12.46.

#### 2.2.2. Synthetic Procedures

##### Graphene Exfoliation [14]

Chlorosulfonic acid-assisted exfoliation of graphite to produce few-layered graphene was realized based on previously reported protocols [14]. A mixture of 0.2 g graphite flakes (>75%, >150 mesh) in 100 mL chlorosulfonic acid was sonicated for 8 h, during temperature fluctuated from 30 °C to 52 °C. The resulting black homogenous dispersion was quenched carefully (highly exothermic reaction) with distilled water. The mixture was filtered through a PTFE membrane filter (pore size 0.2 μm) and washed with water, methanol and dichloromethane. The filter cake was re-dispersed in *N*-methyl-2-pyrrolidone (NMP) (100 mL) with the aid of bath sonication to give a black suspension. Then, the mixture was tip-sonicated (10% power of 150 W, 20 kHz) for 30 min while temperature was kept below 30 °C with the aid of a water-ice bath and the black suspension formed was left at ease overnight. Afterwards, the 2/3 of the black supernatant was collected, filtered through a PTFE membrane filter (pore size 0.2 μm) and washed with water, methanol and dichloromethane. Please note that extreme care should be taken when working with chlorosulfonic acid, since it reacts violently with humidity and water releasing hydrogen chloride gas. A well-ventilated hood is mandatory.

##### Preparation of Pyr-Graphene and Imi-Graphene [15]

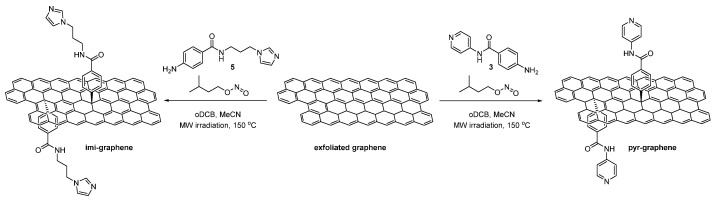



Diazonium salt reaction on graphene was realized by an optimized protocol based on our previously reported procedure [15]. In detail, exfoliated graphene (0.015 g), pyridine (3) (0.150 g, 0.7 mmol) or imidazole (5) (0.171 g, 0.7 mmol) derivative respectively and o-DCB (1 mL) were added in a 10 mL microwave vessel under N_2_ and bath-sonicated for 10 min. Then, MeCN (100 μL) was added, followed by quick addition of isoamyl nitrite (0.429 mL, 3.2 mmol). The vial was sealed with a septum cap and the reaction mixture was microwave (MW) irradiated with 50 Watt for 1 h at 150 ^o^C. After cooling down to room temperature, the reaction mixture was diluted with DMF, filtered over a PTFE membrane filter (pore size 0.2 μm) and sonicated. Τhen, the precipitate was washed repeatedly with large amounts of DMF and CHCl_3_ and MeOH until complete removal of any organic impurities not grafted on the graphene substrate. Pyr-graphene and imi-graphene were then collected after drying under N_2_ as dark grey powders (0.016 g).

##### Preparation of Cobaloxime-Based Materials [Co]-Pyr-Graphene and [Co]-Imi-Graphene

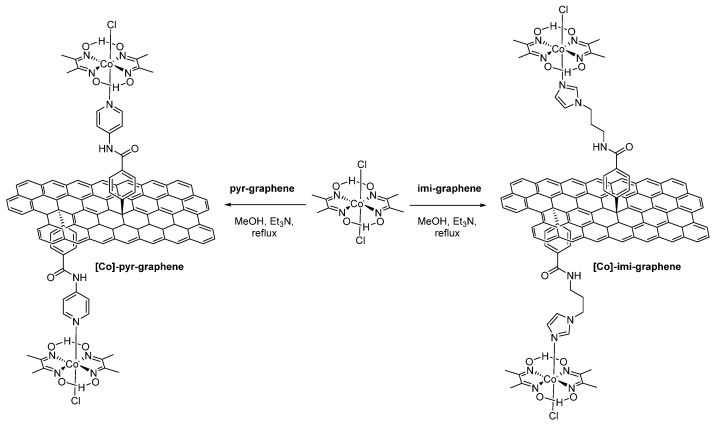



To a stirring suspension of Co(dmgH)_2_Cl_2_ (0.003 g, 0.007 mmol) in MeOH (1 mL), Et_3_N (0.0014 g, 0.014 mmol) was added. The dark green suspension immediately turned brown upon Et_3_N addition and was left stirring for 15 min in r.t. The pyr-graphene or imi-graphene materials (0.01 g) were then added and well-dispersed in MeOH (2 mL). The dispersion was refluxed during a 3-day period. The reaction mixture was then filtered over a PTFE membrane filter (pore size 0.2 μm) and washed with MeOH. The solid residue was sonicated in MeOH, filtered and washed again with large amounts of MeOH to remove organic impurities, dried under N_2_ and collected as a dark grey powder (0.011 g).

## 3. Results and Discussion

**Figure 1:**
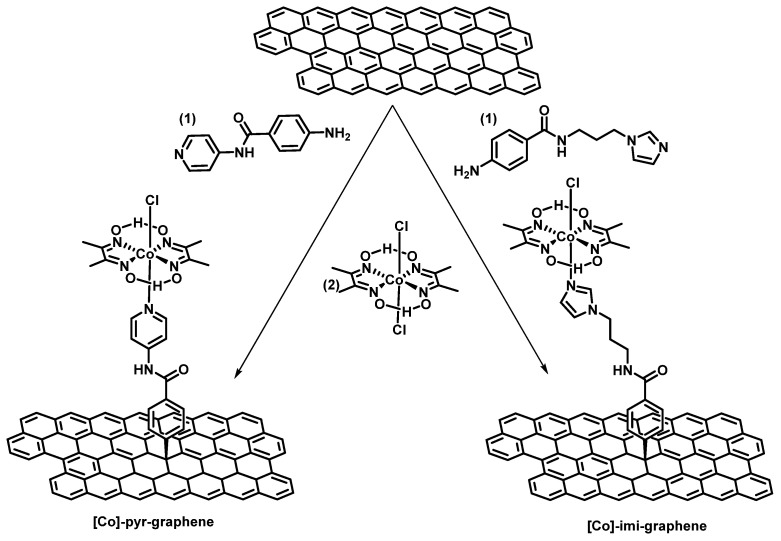
Preparation of [Co]-pyr-graphene and [Co]-imi-graphene materials.

In order to covalently incorporate the desired moieties on the exfoliated graphene lattice, we deployed the successful protocol of diazonium reaction [16]. Both pyridine and imidazole adducts were designed to carry an aryl amine, capable to be converted to the respective diazonium salt under certain conditions and then reduced to the reactive aryl radical [15]. Next, bis(dimethylglyoximato)cobalt(II) complex was employed to furnish the cobaloxime-functionalized [Co]-pyr-graphene and [Co]-imi-graphene (Figure 1).

### 3.1. Raman Spectroscopy

**Figure 2:**
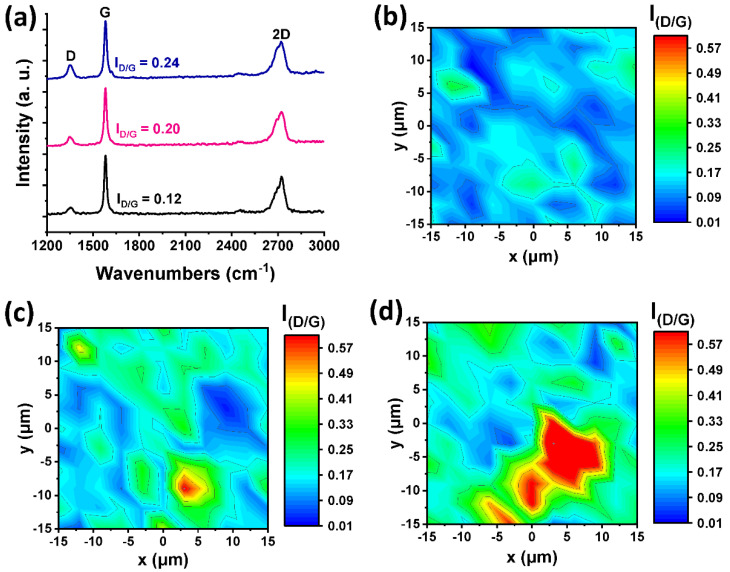
(**a**) Representative (average) Raman spectra (514 nm) of exfoliated graphene (black), [Co]-pyr-graphene (pink) and [Co]-imi-graphene (blue). Spatial Raman spectra (514 nm, 30 × 30 μm areas) of (**b**) exfoliated graphene, (**c**) [Co]-pyr-graphene and (**d**) [Co]-imi-graphene, depicting the I_D_/I_G_ ratio.

**Figure A1:**
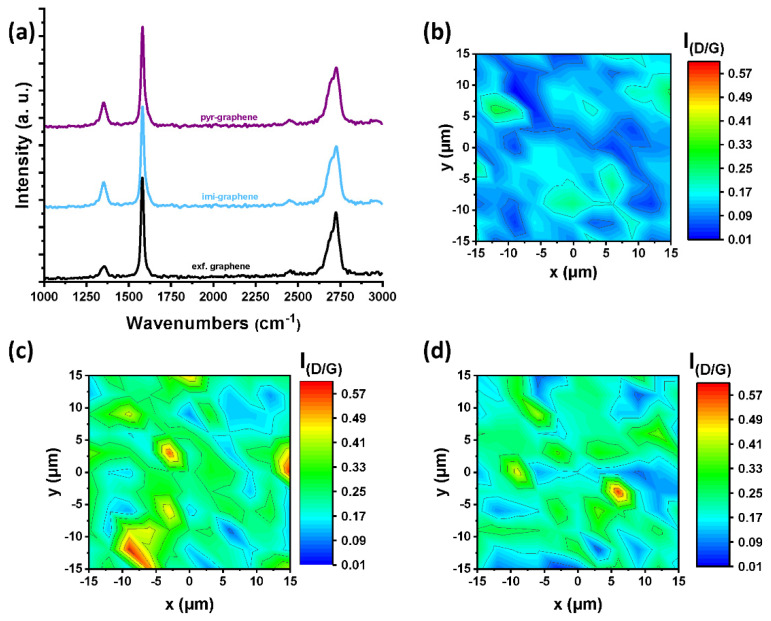
(**a**) Representative (average) Raman spectra (514 nm) of exfoliated graphene (black), pyr-graphene (purple) and imi-graphene (light blue). Spatial Raman spectra (514 nm, 30 × 30 μm area) of (**b**) exfoliated graphene, (**c**) pyr-graphene and (**d**) imi-graphene, depicting the D/G intensity ratio.

Raman spectroscopy unveils information regarding both the lattice structure and functionalization status of graphene-based materials [17,18,19]. Spectral regions of interest are the D (1350 cm^−1^), G (1580 cm^−1^) and 2D (~2720 cm^−1^) bands, which are associated with the defects (sp3 hybridization), lattice integrity of sp2 carbons and stacking order, respectively [18]. Raman spectra (514 nm) of intermediate materials pyr-graphene and imi-graphene are presented in Appendix A Figure A1, along with their corresponding color scale maps of I_D_/I_G_ intensity ratio of 30 × 30 μm areas. The considerable increase in the average D/G intensity ratio value (I_D_/I_G_) from graphene (0.12) to [Co]-pyr-graphene (0.20) and [Co]-imi-graphene (0.24) is indicative of sp^3^ hybridization increase upon covalent grafting [19]. As expected, the I_D_/I_G_ intensity ratio of the intermediate materials remains the same upon their functionalization with the anchoring of cobaloxime moieties for the realization of [Co]-pyr-graphene and [Co]-imi-graphene (Figure 2), since no further interference on the graphene lattice takes place. In Figure 2b–d, the I_D_/I_G_ intensity ratio derived from the corresponding Raman spectra of 30 × 30 μm areas of exfoliated graphene, [Co]-pyr-graphene and [Co]-imi-graphene, are represented as color scale maps. Interestingly, in both functionalized materials the I_D_/I_G_ fluctuates around 0.20 to 0.24 on average, while occasionally surpassing these values, being even 4 times higher than that of exfoliated graphene (0.12). The Raman spectra of exfoliated graphene, [Co]-pyr-graphene and [Co]-imi-graphene (Figure 2a) collectively show the successful covalent functionalization or else chemisorption of pyridine/Co(III) and imidazole/Co(III) coordination compounds on exfoliated graphene’s lattice.

### 3.2. Thermogravimetric Analysis

**Figure 3:**
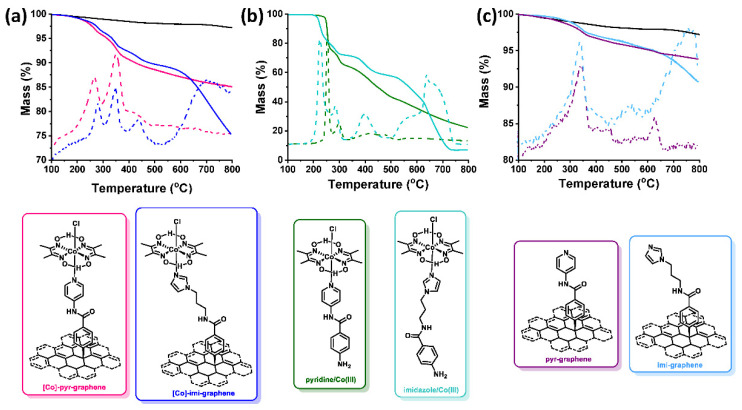
TGA (solid lines) and 1st weight derivative (dashed lines) of (**a**) exfoliated graphene (black), [Co]-pyr-graphene (pink) and [Co]-imi-graphene (blue), (**b**) pyridine/Co(III) (green) and imidazole/Co(III) (teal), and (**c**) exfoliated graphene (black), pyr-graphene (purple) and imi-graphene (light blue). Bottom panel: structure of functionalized materials [Co]-pyr-graphene and [Co]-imi-graphene, model compounds pyridine/Co(III) and imidazole/Co(III), and intermediate materials pyr-graphene and imi-graphene.

Information related to the loading achieved were extracted with the aid of thermogravimetric analysis (TGA) under N_2_ constant flow. While graphene is relatively thermally stable, [Co]-pyr-graphene exhibits an 11.4% mass loss up to 500 °C summarized in two decomposition steps at 264 and 350 °C, as revealed by the 1st order weight derivative (Figure 3a). On the other hand, [Co]-imi-graphene has an 8.3% mass loss up to 500 °C, analyzed in three different decomposition steps at 279, 347 and 436 °C (Figure 3a). In order to gain insight into the functionalization status and degree of each material, model pyridine/Co(III) and imidazole/Co(III) coordination compounds were synthesized and characterized as well (see Experimental section). Specifically, their TGA graphs (Figure 3b) confirm the profile of the functionalized materials (Figure 3a), with sole differences that the thermal events up to 500 °C are shifted to higher temperature for the graphene materials. In detail, for pyridine/Co(III) two decomposition steps are observed at 252 and 297 °C, while for imidazole/Co(III) three steps are registered at 224, 285 and 396 °C This phenomenon is a direct evidence of the successful covalent functionalization, since the covalent attachment of the organic chains on the graphene lattice in comparison to their potential physisorption, has been proved to result in higher temperature decomposition steps [20].

The TGA graphs of pyridine/Co(III) and imidazole/Co(III) compounds are shown in Figure 3b, while those of intermediate materials pyr-graphene and imi-graphene, are depicted in Figure 3c. One decomposition step is registered for each material until 500 °C which is observed in both cases at around 350 °C. Markedly, similar loading is achieved in both cases, as evidenced by the mass loss until 500 °C, that is around 5%, confirming the reproducibility of the diazonium salt reaction on graphene. Due to the vast graphene lattice that causes steric hindrance, to the dispersibility of the material and to the nature of the reaction itself, the coordination step does not proceed quantitatively. Bearing that in mind, a rough estimation of the degrees of functionalization [20] is around 1 pyridine/Co(III) every 330 C atoms and 1 imidazole/Co(III) every 450 C atoms in graphene. The deviation between the two is expected, taking into account the lower yield achieved for the synthesis of imidazole/Co(III) (ca. 52%) model coordination compound, compared to pyridine/Co(III) (ca. 89%).

### 3.3. Electrochemical Characterization

**Figure 4:**
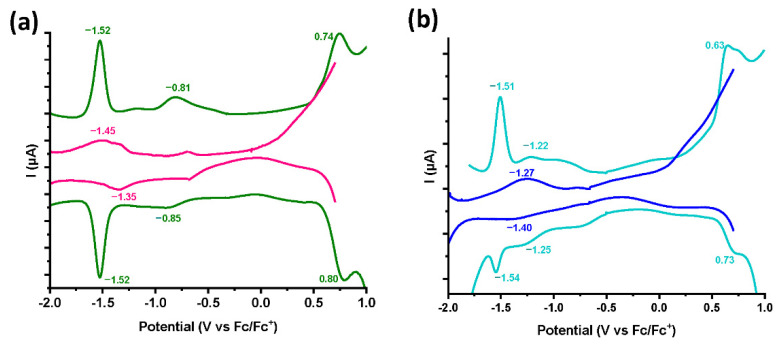
Square wave voltammetry (SWV) assays of (**a**) pyridine/Co(III) (green) and [Co]-pyr-graphene (pink) and (**b**) imidazole/Co(III) (teal) and [Co]-imi-graphene (blue), recorded in N_2_-saturated dry DMF and 0.1 M TBAPF_6_. Pt wire and Pt cloth were used as reference and counter electrode, respectively, while a Pt button electrode was used as the working electrode.

**Figure A2:**
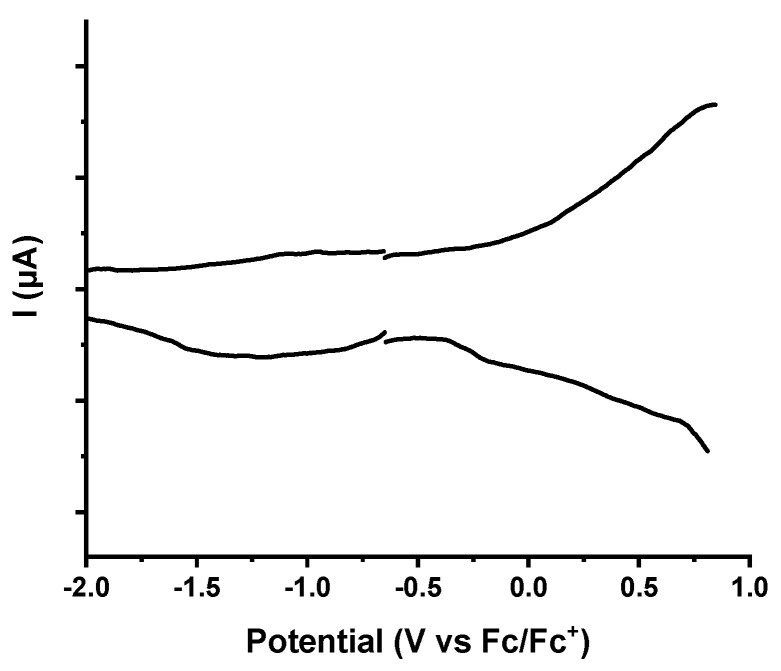
Square wave voltammetry (SWV) assays of exfoliated graphene recorded in N_2_-saturated dry DMF and 0.1 M TBAPF_6_. Pt wire and Pt cloth were used as reference and counter electrode respectively, while a Pt button electrode was used as the working electrode.

Despite the relatively low degree of functionalization, we attempted to confirm the presence of Co(III) via square wave voltammetry recorded in 0.1 M TBAPF_6_ oxygen-free, dry DMF (Figure 4, Table 1). The model compounds imidazole/Co(III) and pyridine/Co(III) were firstly screened and we expected three types of redox couples for each, those being the Co(III)/Co(II), Co(II)/Co(I) and Co(IV)/Co(III) [21,22]. Indeed, for pyridine/Co(III) two completely reversible reductions at −0.83 V and −1.52 V and one reversible oxidation at +0.77 V vs. Fc/Fc^+^ were registered, corresponding to Co(III)/Co(II), Co(II)/Co(I) and Co(IV)/Co(III) redox couples, respectively (Figure 4a) [23]. In parallel, for imidazole/Co(III) we observed two sequential reversible reductions located at −1.24 V and −1.53 V and one reversible oxidation at +0.68 V vs. Fc/Fc^+^ corresponding to Co(III)/Co(II), Co(II)/Co(I) and Co(IV)/Co(III) redox couples, respectively (Figure 4b). Due to the large peak height of the second reduction corresponding to Co(II)/Co(I) redox couple, the first reductive peak of Co(III)/Co(II) couple is masked inside the background current for both model compounds. This is why when screening the functionalized materials, we were able to distinguish only one reversible reduction at −1.39 V vs. Fc/Fc^+^ for [Co]-pyr-graphene and at −1.34 V vs. Fc/Fc^+^ for [Co]-imi-graphene (Figure 4a,b). In Appendix A Figure A2, the square wave voltammetry graph of exfoliated graphene is provided as reference and as expected, no oxidation or reduction peaks are registered.

The oxidation from Co(III) to Co(IV), being close to the upper limit of the electrochemical window of DMF, was also not visible in functionalized materials, which is the case for most studies on molecular cobaloximes [9,21]. However, the main parameter responsible for the loss of these signals is linked to the nature of the materials, which behave completely differently from their molecular analogues. The reductive peaks appear broadened and are thus poorly defined. Moreover, they shifted towards more positive potential values by around 0.15 V on average, compared to the E_1/2_ of Co(II)/Co(I) reductive couple of their molecular analogues. Especially, the easier Co(II)/Co(I) reduction, revealed by the positive shift on the E_1/2_ for both [Co]-pyr-graphene and [Co]-imi-graphene, is in line with the higher electron donation ability channeled by graphene.

### 3.4. Imaging

**Figure 5:**
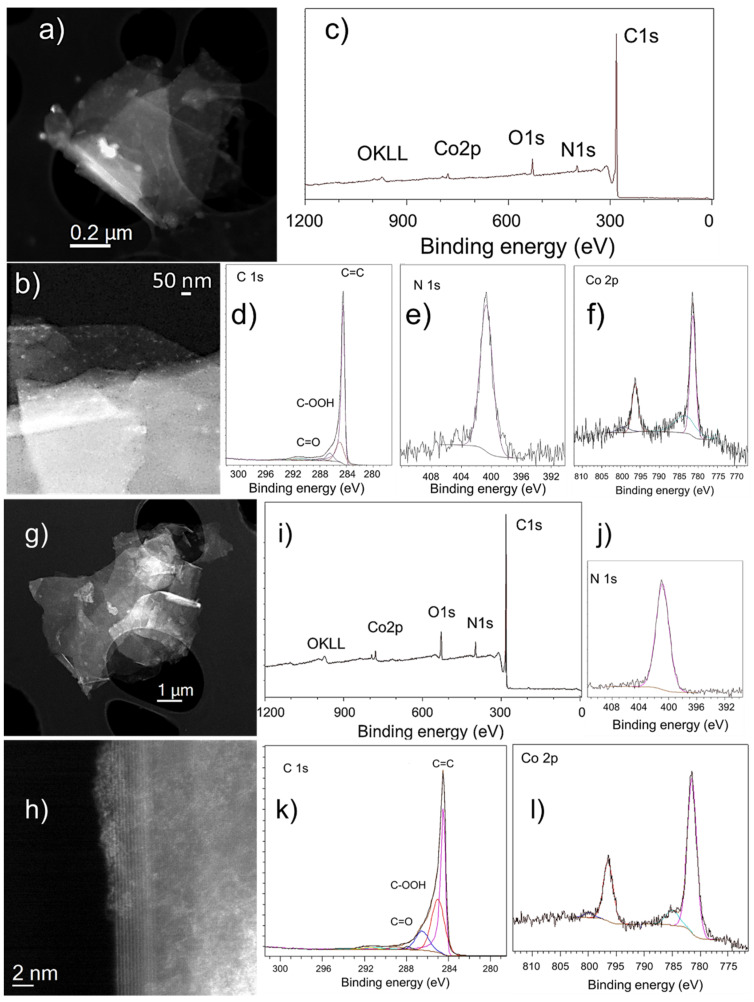
(**a,b**) and (**g,h**) HAADF-STEM (low-magnification (**a,g**), high-magnification (**b,h**)) micrographs of [Co]-pyr-graphene and [Co]-imi-graphene, respectively. (**c**) and (**i**) XPS survey spectra of [Co]-pyr-graphene and [Co]-imi-graphene, respectively. (**d–f**) and (**j–l**) HR-XPS spectra of [Co]-pyr-graphene and [Co]-imi-graphene, respectively.

Combination of local scanning transmission electron microscopy (STEM) studies and macroscopic X-ray photoelectron spectroscopy (XPS) analyses constitute perfect approaches for getting important information on structural and chemical composition, even at subnanometer scale, of complex systems. We performed such studies of [Co]-pyr-graphene and [Co]-imi-graphene. Figure 5a,g display low-magnification micrographs, where the graphene flakes are observed. From the high-angle annular dark-field (HAADF) high-resolution STEM images (Figure 5b,h) some deposits corresponding to the [Co]-pyr and [Co]-imi moieties can be distinguished.

**Figure 6:**
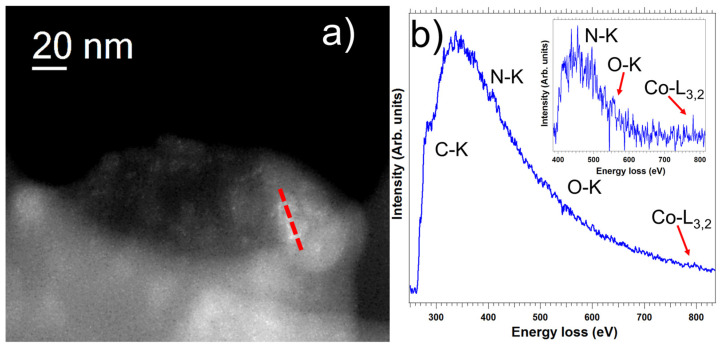
(**a**) HAADF-STEM micrograph of [Co]-pyr-graphene recorded on one graphene flake. An EELS spectrum-line has been acquired on the red marked line. (**b**) The EEL spectrum corresponds to the sum of 20 EEL spectra recorded in the red line. The C-K, N-K, O-K and Co-L_3,2_ edges are clearly observed.

**Figure 7:**
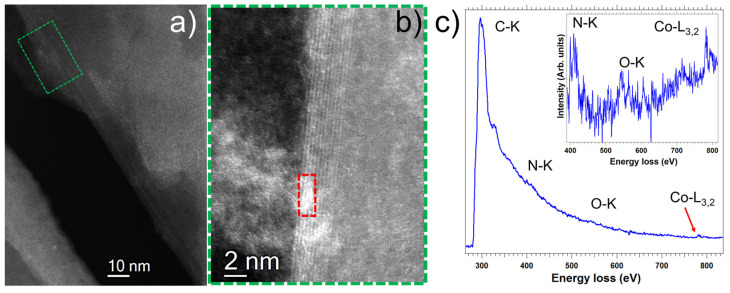
(**a,b**) HAADF-STEM micrographs of [Co]-imi-graphene recorded on one graphene flake. An EELS spectrum-image has been acquired on the red marked area highlighted. (**c**) The EEL spectrum corresponds to the sum of 24 EEL spectra recorded in this red marked area. The C-K, N-K, O-K and Co-L_3,2_ edges are clearly observed.

Further electron-energy loss spectroscopy (EELS) analyses confirm these findings (Figure 6 and Figure 7). Furthermore, from the XPS analyses, the chemistry of these heterostructures was investigated. Figure 5c,i show survey XPS spectra for [Co]-pyr-graphene and [Co]-imi-graphene, respectively, where the existence of C, N, O and Co is confirmed. High-resolution spectra of these elements were acquired and also depicted in this figure (except for the O as no further information is extracted from the O 1s spectra). Figure 5d,k display the C 1s part of the XPS spectrum, with the peaks at 284.5, ~285, 286.4 and 288 eV corresponding to C=C and C-C, COOH and C=O, respectively. The N 1s is depicted at Figure 5e,i and corresponds to the pyridine unit. Finally, in Figure 5f,l, the Co 2p is observed. All these findings allow us to understand and justify the configuration of these hybrids.

### 3.5. Catalytic Studies on the Hydrogen Evolution Reaction

**Figure 8:**
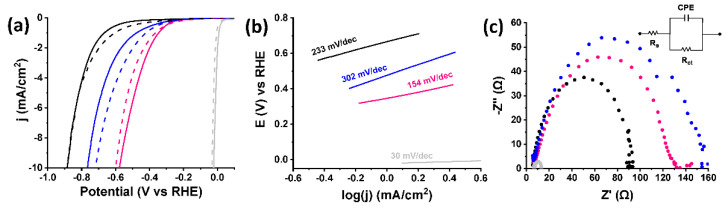
(**a**) LSV polarization curves for HER before (solid lines) and after (dashed lines) 10,000 catalytic cycles, and (**b**) Tafel slope, of exfoliated graphene (black), [Co]-pyr-graphene (pink), [Co]-imi-graphene (blue) and Pt/C (light grey). The LSVs were obtained at 1600 rpm rotation speed and 5 mV/s scan rate in N_2_-saturated aqueous 0.5 M H_2_SO_4_. The current densities are normalized to the geometric electrode area. (**c**) Nyquist plots of [Co]-pyr-graphene (pink), [Co]-imi-graphene (blue), exfoliated graphene (black) and benchmarck HER electrocatalyst Pt/C (light grey). Inset: Equivalent circuit used for fitting the EIS data. Rs refers to the overall series resistance, Rct to the charge transfer resistance and CPE to the constant phase angle element, which represents the double layer capacitance of solid electrode in a realistic situation. The EIS measurements (Nyquist plots) were recorded at the low overpotential (kinetic) region.

**Figure A3:**
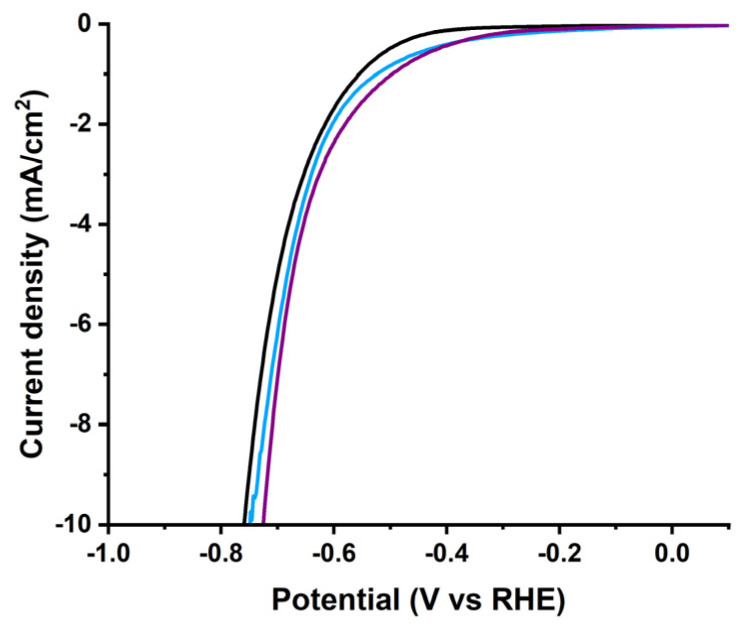
LSV polarization curves for HER of exfoliated graphene (black), pyr-graphene (purple) and imi-graphene (light blue). The LSVs were recorded at 1600 rpm rotation speed and 5 mV/s scan rate in N_2_-saturated aqueous 0.5 M H_2_SO_4_. The current densities are normalized to the geometric electrode area.

Having secured the structural characterization of the functionalized materials, we proceeded with the electrocatalytic HER investigation. Cobaloxime-modified graphene analogues [Co]-pyr-graphene and [Co]-imi-graphene as well as their precursor, exfoliated graphene, were assessed towards HER in aqueous 0.5 M H_2_SO_4_ electrolyte. Figure 8a depicts the corresponding LSV polarization curves in comparison with benchmark HER electrocatalyst Pt/C. While reference materials pyr-graphene and imi-graphene do not differentiate from exfoliated graphene (Figure A3) as expected, both cobaloxime-functionalized materials exhibit more positive onset potential than their precursors by around 200–300 mV, validating our initial hypothesis. In detail, graphene’s onset potential is −0.59 V, while [Co]-imi-graphene’s is −0.38 V and [Co]-pyr-graphene’s appears even closer to Pt/C, at −0.29 V vs. RHE. In line with standards of the research community, which has adopted the overpotential at −10 mA/cm^2^ (*η_10_*) (in analogy with the standards set for solar to fuel energy conversion devices) [24] as activity marker in electrocatalytic HER, we report in Table 2 the respective values for our study. [Co]-pyr-graphene exhibits the lowest overpotential at −10 mA/cm^2^ (−0.57 V), almost 200 mV lower than [Co]-imi-graphene’s (e.g., −0.76 V) and more than 300 mV lower compared to exfoliated graphene’s (e.g., −0.88 V). To our pleasant surprise, [Co]-pyr-graphene and [Co]-imi-graphene differ significantly, with the former being drastically superior. This is confirmed also based on the inherent kinetics of each material as reflected by the Tafel analysis (Figure 8b). In fact, [Co]-imi-graphene exhibits the highest Tafel slope of 302 mV/dec, followed by exfoliated graphene with a value of 203 mV/dec, while the lowest one of 153 mV/dec corresponds to [Co]-pyr-graphene. The lower the Tafel slope value, the faster the charge transfer across the electrocatalyst interface, which is the case for [Co]-pyr-graphene. Surprisingly, whereas the two functionalized materials differentiate solely on the axial ligation (pyridine vs. imidazole functional group), [Co]-imi-graphene exhibits double the Tafel value of [Co]-pyr-graphene, meaning the kinetics of the water dissociation step are efficiently facilitated on the surface of the latter [25]. Further insight on this matter is provided by electrochemical impedance spectroscopy (EIS). The Nyquist plots, which were fitted by using an equivalent circuit, are depicted in Figure 8c. The charge-transfer resistance (R_ct_) value of 116 Ω for [Co]-pyr-graphene was lower than that of 133 Ω for [Co]-imi-graphene albeit higher than exfoliated graphene (e.g., 87 Ω). The lower R_ct_ value accounts for a faster Faradaic process and, therefore, superior HER kinetics [26]. The higher R_ct_ for both modified materials compared to graphene can be probably attributed to the disruption of the vast sp^2^ graphene lattice or else modulation of its electronic structure due to surface covalent modification and immobilization of cobaloxime. Still, the pyridine analogue surpasses kinetically the imidazole analogue, validating the rest electrocatalytic parameters (Table 2).

**Figure A4:**
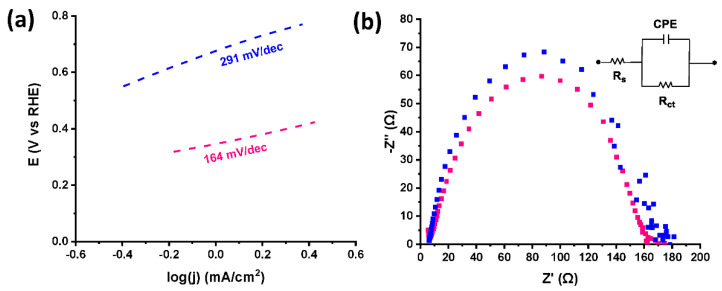
(**a**) Tafel slope [Co]-pyr-graphene (pink) and [Co]-imi-graphene (blue) after 10,000 catalytic cycles. The LSVs were obtained at 1600 rpm rotation speed and 5 mV/s scan rate in N_2_-saturated aqueous 0.5 M H_2_SO_4_. The current densities are normalized to the geometric electrode area. (**b**) Nyquist plots of [Co]-pyr-graphene (pink) and [Co]-imi-graphene (blue) after 10,000 catalytic cycles. Inset: Equivalent circuit used for fitting the EIS data. Rs refers to the overall series resistance, Rct to the charge transfer resistance and CPE to the constant phase angle element, which represents the double layer capacitance of solid electrode in a realistic situation. The EIS measurements (Nyquist plots) were recorded at the low overpotential (kinetic) region.

In contrast, durability studies reflect a different point of view. The LSV polarization curves of the graphene materials after 10,000 catalytic cycles are presented in Figure 8a. Notably, while exfoliated graphene preserves its activity upon recycling, due to its mechanical stability, an amelioration of HER catalytic performance is observed for [Co]-imi-graphene, as reflected by the 50 mV lower overpotential value at −10 mA/cm^2^ (Table 2). Evidently, [Co]-imi-graphene has a remarkable long-term stability and actually equilibrates at an improved onset potential of −0.34 V vs. RHE upon recycling. On the other hand, an extra 20 mV is required for [Co]-pyr-graphene to reach −10 mA/cm^2^ after 10,000 catalytic cycles. Given the common instability of the cobaloxime ring [27] that reflects also on non-covalent cobaloxime/graphene heterostructures [28], this result is a solid evidence of the positive effect of the covalent cobaloxime grafting on the lifetime of the electrocatalysts in acidic HER conditions. The stability of the prepared catalysts upon extensive cycling is further validated by the Tafel analysis performed after 10,000 catalytic cycles, as well as by the EIS measurements after recycling (Table 2). In Appendix A Figure A4, the Tafel slopes of [Co]-pyr-graphene and [Co]-imi-graphene and the respective Nyquist plots after recycling are depicted. Notably, the Tafel slopes are only slightly affected (negatively for the pyridine analogue and positively for the imidazole one), while the charge-transfer resistance (R_ct_) of both is faintly enlarged, mirroring vaguely inferior kinetics after 10,000 catalytic cycles. The plausible protonation of the imidazole ring in the highly acidic pH conditions, decreases the Lewis basicity of imidazole ligand, resulting in less electron-donating activity, thus difficult protonation of the Co-H intermediate species that eventually releases H_2_ [8,29]. This is not the case for pyridine however. Therefore, this reasoning could explain the electrocatalytic superiority induced by pyridine axial coordination. In contrast, the relatively stronger Co-N axial ligation of imidazole, induced by its higher basicity, is probably responsible for the elevated long-term performance.

## 4. Conclusions

In summary, despite the prima facie evidence, axial ligation of the cobaloxime functionalized graphene plays a major role in the HER electrocatalytic process. Pyridine axial ligand induced a better electrocatalytic activity, which was also confirmed kinetically. In contrast, imidazole moiety was found to equilibrate long-term and resist any deactivation upon extensive cycling. Our results highlight the importance of the primary axial coordination sphere substituents to cobaloxime complexes utilized as HER electrocatalysts and provide a different point of view to researchers engaging in the surface engineering of 2D nanomaterials as electrocatalysts.

## Figures and Tables

**Table 1:** Square wave voltammetry (SWV) data for pyridine/Co(III), imidazole/Co(III), [Co]-pyr-graphene and [Co]-imi-graphene recorded in N_2_-saturated 0.1 M TBAPF_6_ in dry DMF.

Material/Compound	Co(III)/Co(II) E_1/2_^Red^ (ΔEpa,pc)/ V vs. Fc/Fc^+^	Co(II)/Co(I) E_1/2Red_ (ΔEpa,pc)/ V vs. Fc/Fc^+^	Co(IV)/Co(III) E_1/2_^Ox^ (ΔEpa,pc)/ V vs. Fc/Fc^+^
Pyridine/Co(III)	−0.83 (0.04)	−1.52 (0.0)	0.77 (0.06)
Imidazole/Co(III)	−1.24 (0.06)	−1.53 (0.03)	0.68 (0.1)
[Co]-pyr-graphene	-	−1.39 (0.1)	-
[Co]-imi-graphene	-	−1.34 (0.13)	-

**Table 2:** Electrocatalytic HER parameters for exfoliated graphene, functionalized materials [Co]-pyr-graphene and [Co]-imi-graphene and benchmark HER electrocatalyst Pt/C.

Electrocatalyst	Onset PoAPPential (V vs. RHE)		Potential @ −10 mA/cm^2^ (V vs. RHE)		Tafel Slope (mV/dec)		R_ct_ (Ω)	
	0 c *	10,000 c *	0 c *	10,000 c *	0 c *	10,000 c *	0 c *	10,000 c *
exfoliated graphene	−0.588	−0.497	−0.887	−0.887	233	-	87	-
[Co]-pyr-graphene	−0.296	−0.299	−0.576	−0.598	154	164	116	122
[Co]-imi-graphene	−0.377	−0.342	−0.766	−0.717	302	291	133	141
Pt/C	0.02	0.01	−0.03	−0.03	30	-	6	-

* Before (0 c) and after (10,000 c) 10,000 catalytic cycles.

## Data Availability

Not applicable.

## Supplementary Materials

The following supplementary material can be downloaded at: https://www.mdpi.com/article/10.3390/nano12173077/s1, Figures S1–S10: ^1^H and ^13^C NMR data of synthesized compounds.

## References

[1] Le Goff A., Artero V., Jousselme B., Tran P.D., Guillet N., Métayé R., Fihri A., Palacin S., Fontecave M. (2009). From hydrogenases to noble metal-free catalytic nanomaterials for H_2_ production and uptake. Science.

[2] Dolui D., Khandelwal S., Majumder P., Dutta A. (2020). The odyssey of cobaloximes for catalytic H_2_ production and their recent revival with enzyme-inspired design. Chem. Commun..

[3] Sowmya S., Vijaikanth V. (2022). Electrochemistry and electrocatalytic activity of cobaloxime complexes. ChemistrySelect.

[4] Andreidis E.S., Jacques P.-A., Tran P.D., Leyris A., Chavarot-Kerlidou M., Jousselme B., Matheron M., Pecaut J., Palacin S., Fontecave M. (2013). Molecular engineering of a cobalt-based electrocatalytic nanomaterial for H₂ evolution under fully aqueous conditions. Nat. Chem..

[5] Kaeffer N., Morozan A., Artero V. (2015). Oxygen tolerance of a molecular engineered cathode for hydrogen evolution based on a cobalt diimine–dioxime catalyst. J. Phys. Chem. B.

[6] Kaeffer N., Morozan A., Fize J., Martinez E., Guetaz L., Artero V. (2016). The dark side of molecular catalysis: Diimine–dioxime cobalt complexes are not the actual hydrogen evolution electrocatalyst in acidic aqueous solutions. ACS Catal..

[7] Reuillard B., Warnan J., Leung J.J., Wakerley D.W., Reisner E. (2016). A poly(cobaloxime)/carbon nanotube electrode: Freestanding buckypaper with polymer-enhanced H_2_-evolution performance. Angew. Chem. Int. Ed..

[8] Wakerley D.W., Reisner E. (2014). Development and understanding of cobaloxime activity through electrochemical molecular catalyst screening. Phys. Chem. Chem. Phys..

[9] Panagiotopoulos A., Ladomenou K., Sun D., Artero V., Coutsolelos A.G. (2016). Photochemical hydrogen production and cobaloximes: The influence of the cobalt axial N-ligand on the system stability. Dalton Trans..

[10] Mu F., Coffing S.L., Riese D.J., Geahlen R.L., Verdier-Pinard P., Hamel E., Johnson J., Cushman M. (2001). Design, synthesis, and biological evaluation of a series of lavendustin A analogues that inhibit EGFR and syk tyrosine kinases, as well as tubulin polymerization. J. Med. Chem..

[11] Samadi S., Jadidi K., Khanmohammadi B., Tavakoli N. (2016). Heterogenization of chiral mono oxazoline ligands by grafting onto mesoporous silica MCM-41 and their application in copper-catalyzed asymmetric allylic oxidation of cyclic olefins. J. Catal..

[12] De Vita D., Angeli A., Pandolfi F., Bortolami M., Costi R., Di Santo R., Suffredini E., Ceruso M., Del Prete S., Capasso C. (2017). Inhibition of the α-carbonic anhydrase from Vibrio cholerae with amides and sulfonamides incorporating imidazole moieties. J. Enzyme Inhib. Med. Chem..

[13] Lazarides T., Peuntinger K., Dafnomili D., Charalambidis G., Landrou G., Kahnt A., Sabatini R., McCamant D.W., Gryko D.T., Coutsolelos A.G. (2013). Photoinduced charge transfer in porphyrin–cobaloxime and corrole–cobaloxime hybrids. J. Phys. Chem. C.

[14] Stergiou A., Sideri I.K., Kafetzi M., Ioannou A., Arenal R., Mousdis G., Pispas S., Tagmatarchis N. (2022). Methylammonium lead bromide perovskite nano-crystals grown in a poly[styrene-co-(2-(dimethylamino)ethyl methacrylate)] matrix immobilized on exfoliated graphene nano-sheets. Nanomaterials.

[15] Pagona G., Zervaki G.E., Sandanayaka A.S.D., Ito O., Charalambidis G., Hasobe T., Coutsolelos A.G., Tagmatarchis N. (2012). Carbon Nanohorn–Porphyrin Dimer Hybrid Material for Enhancing Light-Energy Conversion. J. Phys. Chem. C.

[16] Paulus G.L.C., Wang Q.H., Strano M.S. (2013). Covalent electron transfer chemistry of graphene with diazonium salts. Acc. Chem. Res..

[17] Graf D., Molitor F., Ensslin K., Stampfer C., Jungen A., Hierold C., Wirtz L. (2007). Spatially resolved raman spectroscopy of single- and few-layer graphene. Nano Lett..

[18] Wu J.-B., Lin M.-L., Cong X., Liu H.-N., Tan P.-H. (2018). Raman spectroscopy of graphene-based materials and its applications in related devices. Chem. Soc. Rev..

[19] Koehler F.M., Jacobsen A., Ensslin K., Stampfer C., Stark W.J. (2010). Selective chemical modification of graphene surfaces: Distinction between single- and bilayer graphene. Small.

[20] Schirowski M., Hauke F., Hirsch A. (2019). Controlling the degree of functionalization: In-depth quantification and side-product analysis of diazonium chemistry on SWCNTs. Chem. Eur. J..

[21] Michael Elliott C., Hershenhart E., Finke R.G., Smith B.L. (1981). Coenzyme B12 model studies: An electrochemical comparison of both alkylcobaloxime and nonalkyl cobaloxime and Co[C2(DO)(DOH)pn] complexes to coenzyme B12. J. Am. Chem. Soc..

[22] Ngameni E., Ngoune J., Nassi A., Belombe M.M., Roux R. (1996). Electrochemical studies and electronic spectroscopic examination of some cobaloximatic complexes based on 2,3-butanedione dioxime or dimethylglyoxime with identical or mixed axial ligands. Electrochim. Acta.

[23] Mandal D., Gupta B.D. (2005). Cobaloximes with dimesitylglyoxime:  Synthesis, characterization, and spectral correlations with the related cobaloximes. Organometallics.

[24] Anantharaj S., Karthik P.E., Noda S. (2021). The significance of properly reporting turnover frequency in electrocatalysis research. Angew. Chem. Int. Ed..

[25] Shinagawa T., Garcia-Esparza A.T., Takanabe K. (2015). Insight on Tafel slopes from a microkinetic analysis of aqueous electrocatalysis for energy conversion. Sci. Rep..

[26] Vrubel H., Moehl T., Gratzel M., Hu X. (2013). Revealing and accelerating slow electron transport in amorphous molybdenum sulphide particles for hydrogen evolution reaction. Chem. Commun..

[27] Donck S., Fize J., Gravel E., Doris E., Artero V. (2016). Supramolecular assembly of cobaloxime on nanoring-coated carbon nanotubes: Addressing the stability of the pyridine–cobalt linkage under hydrogen evolution turnover conditions. Chem. Commun..

[28] Soliman A.B., Haikal R.R., Abugableac A.A., Alkordi M.H. (2017). Microporous cobaloxime–graphene composite: A reloadable non-noble metal catalyst platform for the proton reduction reaction. J. Mater. Chem. A.

[29] Razavet M., Artero V., Fontecave M. (2005). Proton Electroreduction Catalyzed by Cobaloximes:  Functional Models for Hydrogenases. Inorg. Chem..

