# Supplementary material for: Pyridine vs. Imidazole Axial Ligation on Cobaloxime Grafted Graphene: Hydrogen Evolution Reaction Insights
Source: Nanomaterials (Basel). 2022 Sep 5;12(17):3077. doi: 10.3390/nano12173077 (PMC9458012; doi:10.3390/nano12173077)
Supplement: Supplementary file 1 [file nanomaterials-12-03077-s001.zip › nanomaterials-1891115-supplementary.pdf]

nanomaterials

*Supplementary Material*

# Pyridine vs. Imidazole Axial Ligation on Cobaloxime Grafted Graphene: Hydrogen Evolution Reaction Insights

**Ioanna K. Sideri [1], Georgios Charalambidis [2], Athanassios G. Coutsolelos [2], Raul Arenal [3,4,5] and Nikos Tagmatarchis [1,*]**

[1] Theoretical and Physical Chemistry Institute, National Hellenic Research Foundation, 11635 Athens, Greece
[2] Chemistry Department, Laboratory of BioInorganic Chemistry, University of Crete, 710 03 Heraklion, Greece
[3] Laboratorio de Microscopias Avanzadas (LMA), Universidad de Zaragoza, 50018 Zaragoza, Spain
[4] Instituto de Nanociencia y Materiales de Aragon (INMA), CSIC-U. de Zaragoza, 50009 Zaragoza, Spain
[5] ARAID Foundation, 50018 Zaragoza, Spain
* Correspondence: tagmatar@eie.gr

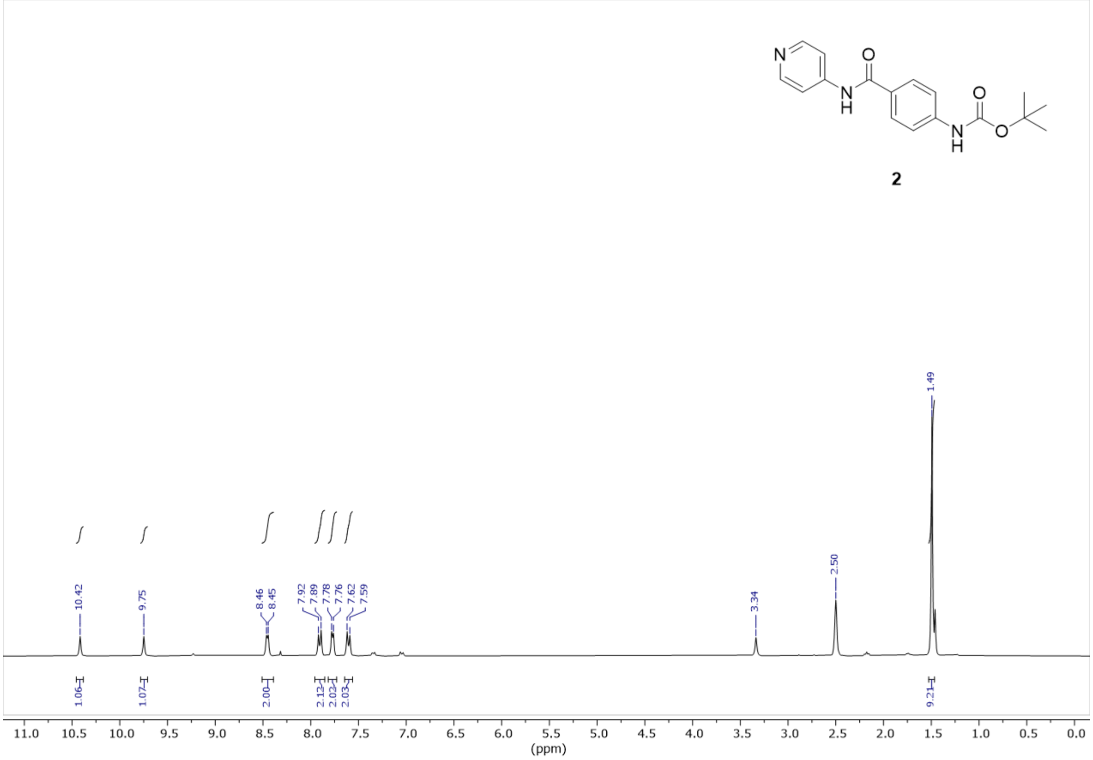

**Figure S1.** $^1$H NMR (300 MHz, DMSO-$d_6$) spectrum of compound **2**.

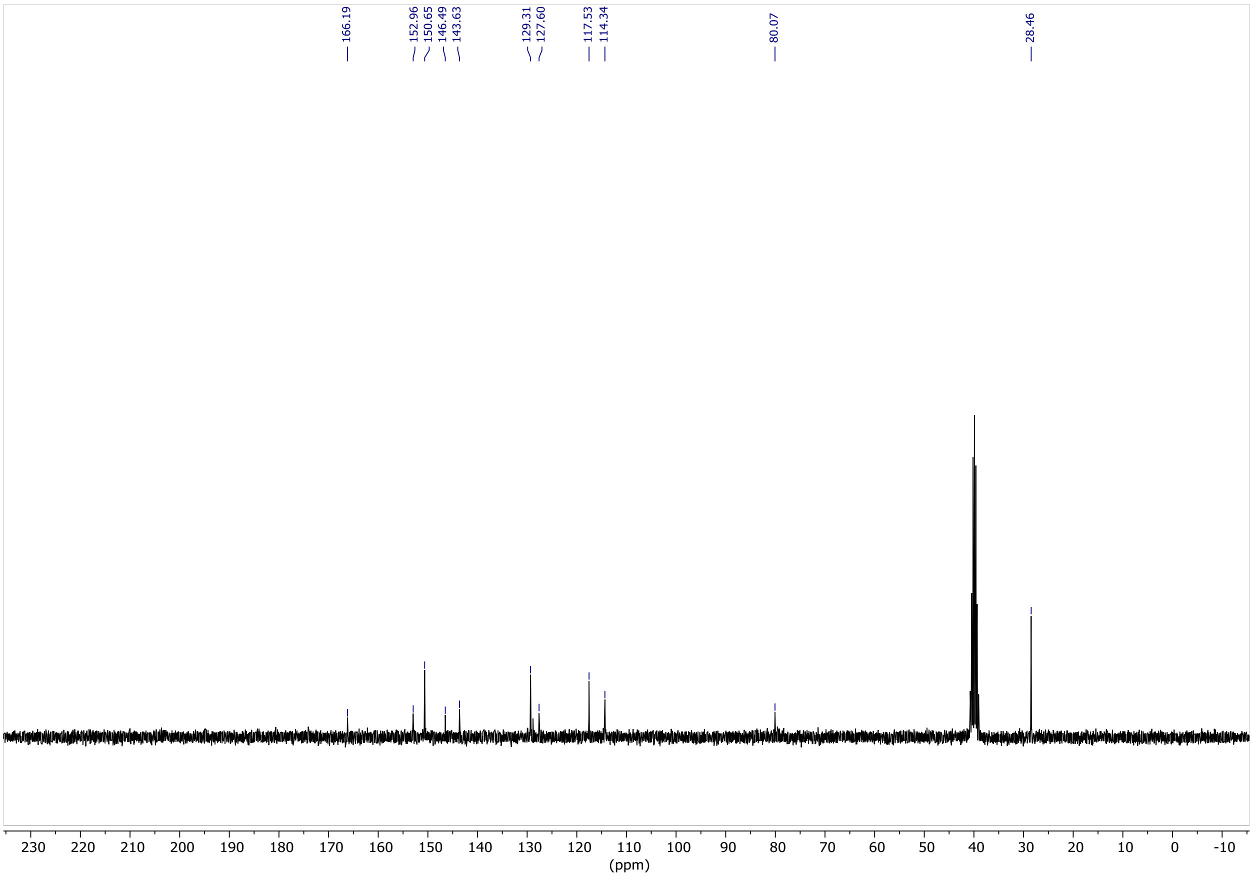


**Figure S2.** $^{13}C\{1\}$ NMR (75.5 MHz, DMSO-$d_6$) spectrum of compound **2**.

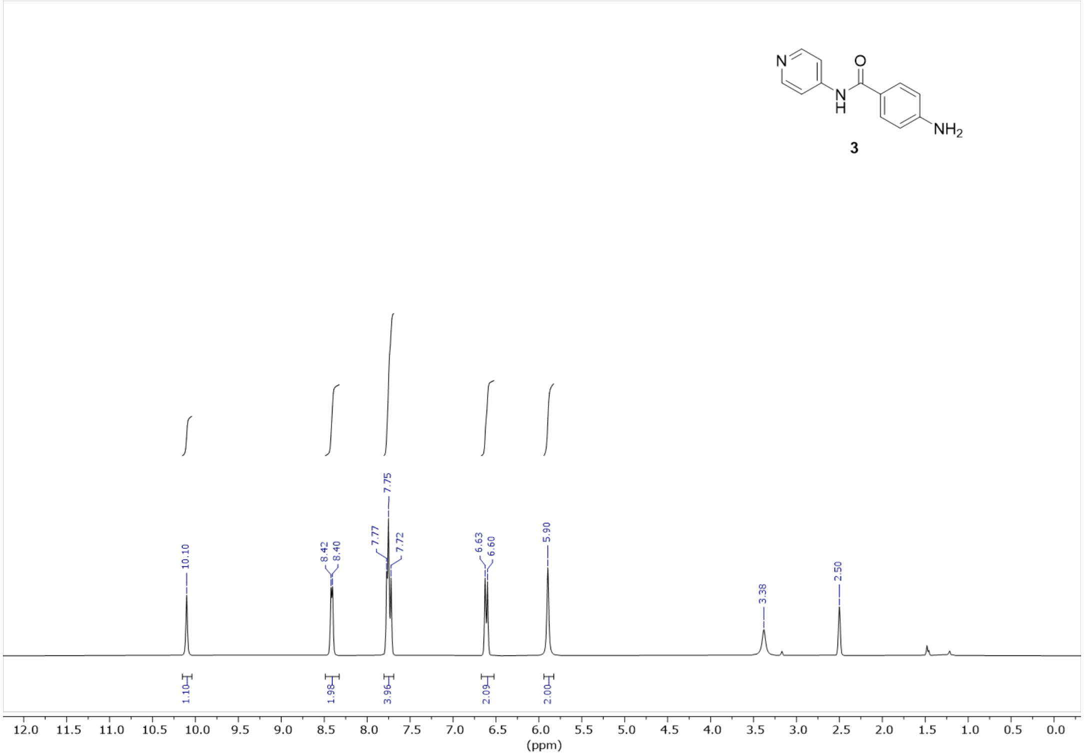


**Figure S3.** $^{1}H$ NMR (300 MHz, DMSO-$d_6$) spectrum of compound **3**.

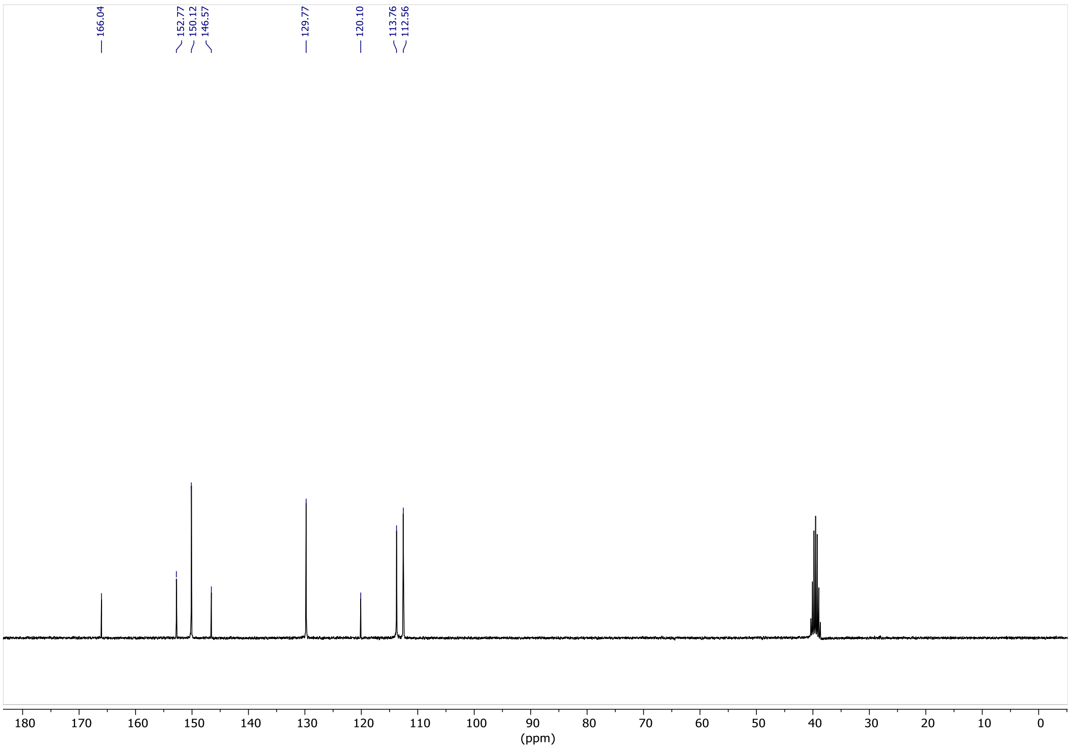


**Figure S4.** $^{13}C\{1\}$ NMR (75.5 MHz, DMSO-$d_6$) spectrum of compound **3**.

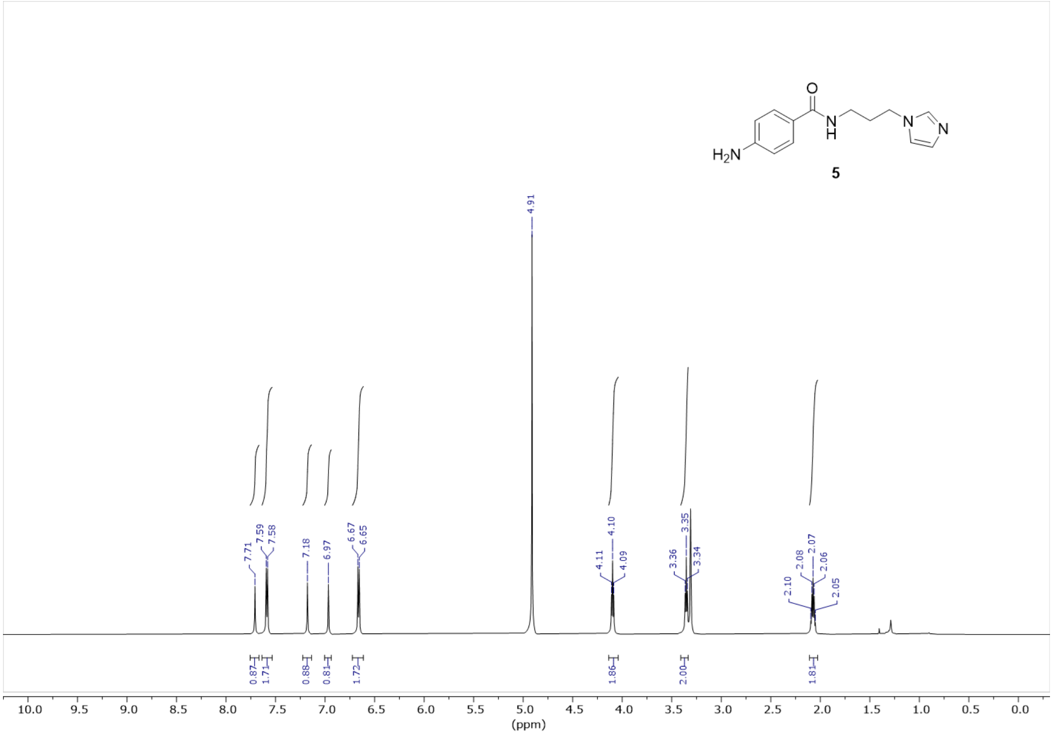


**Figure S5.** $^{1}H$ NMR (600 MHz, $CD_3OD$-$d_4$) spectrum of compound **5**.

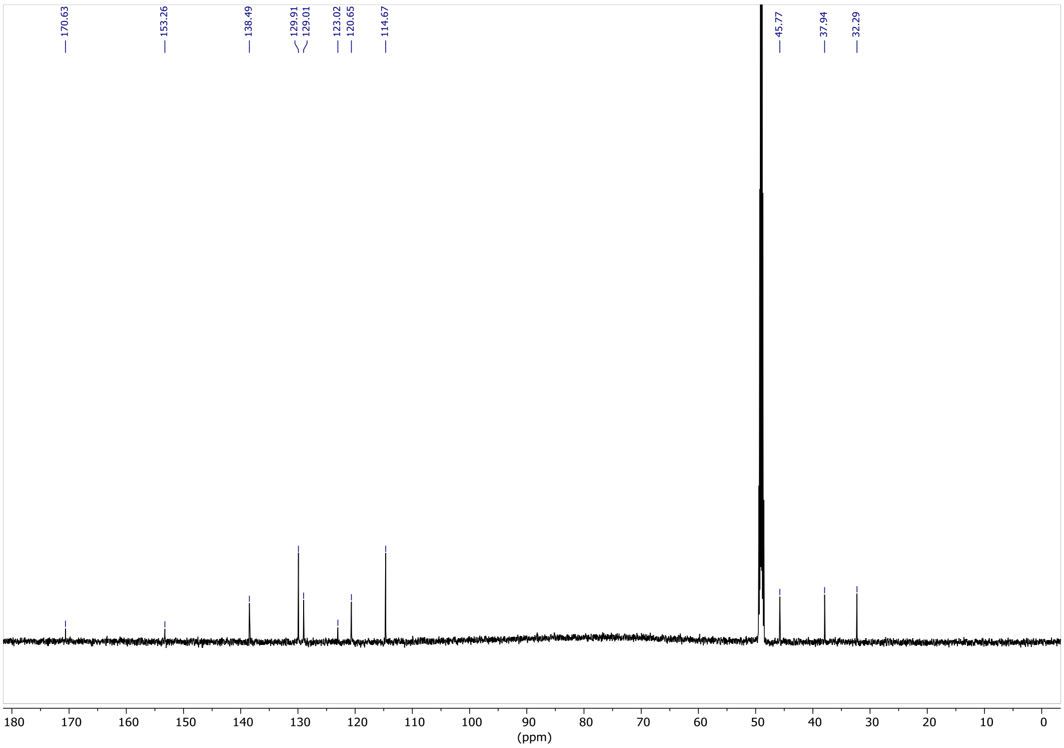


**Figure S6.** $^{13}C\{1\}$ NMR (150.9 MHz, $CD_3OD$-*d*4) spectrum of compound **5**.

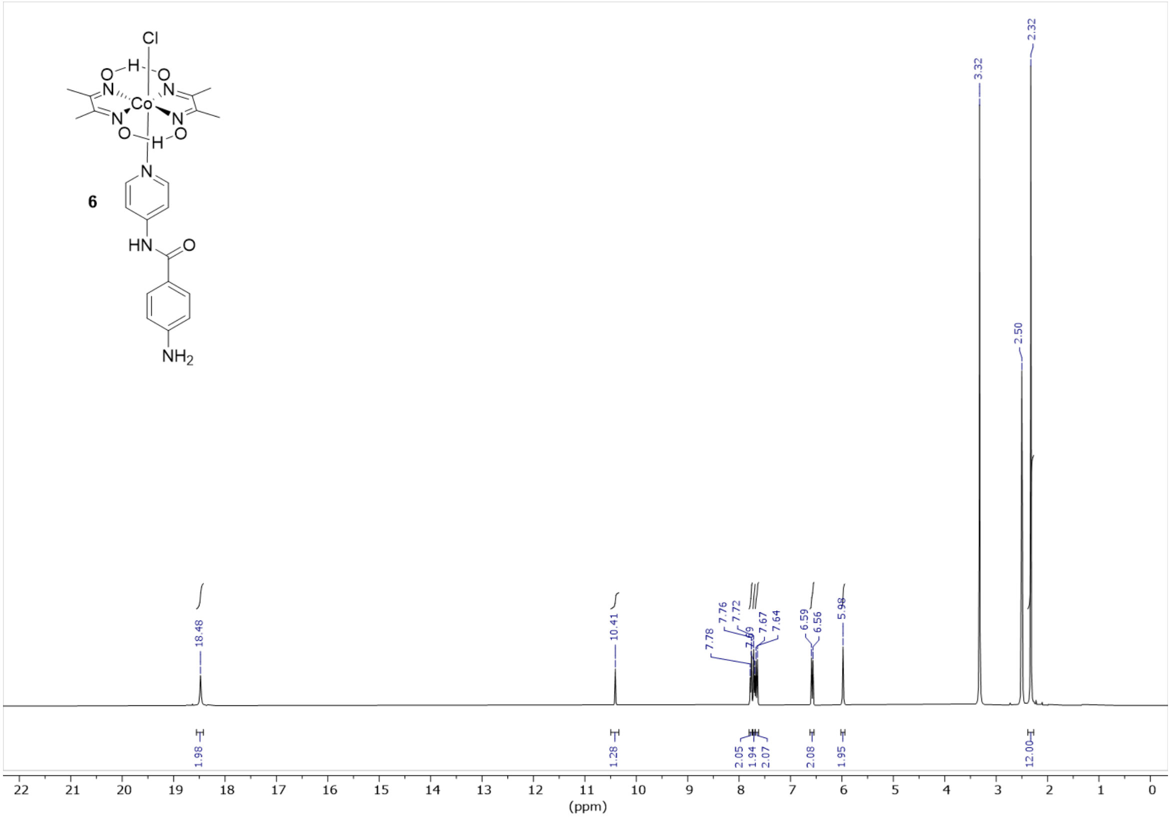


**Figure S7.** $^{1}H$ NMR (600 MHz, DMSO-*d*6) spectrum of compound **6**.

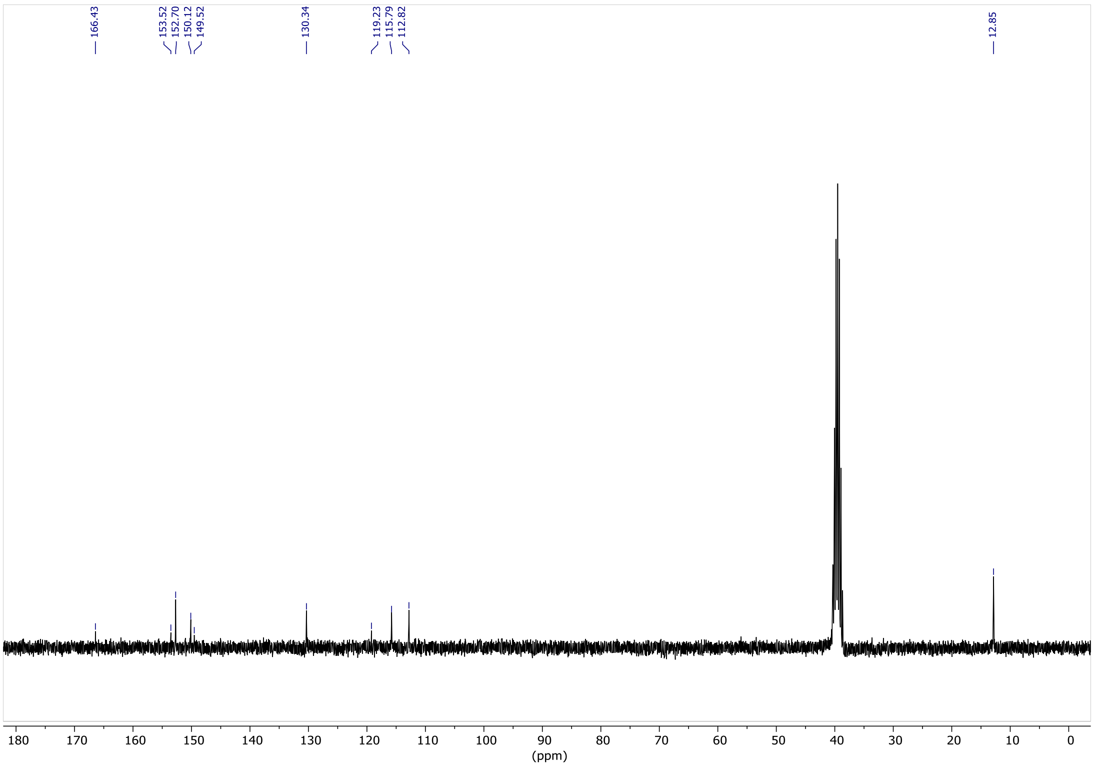


**Figure S8.** $^{13}$C{1} NMR (150.9 MHz, DMSO-$d_6$) spectrum of compound **6**.

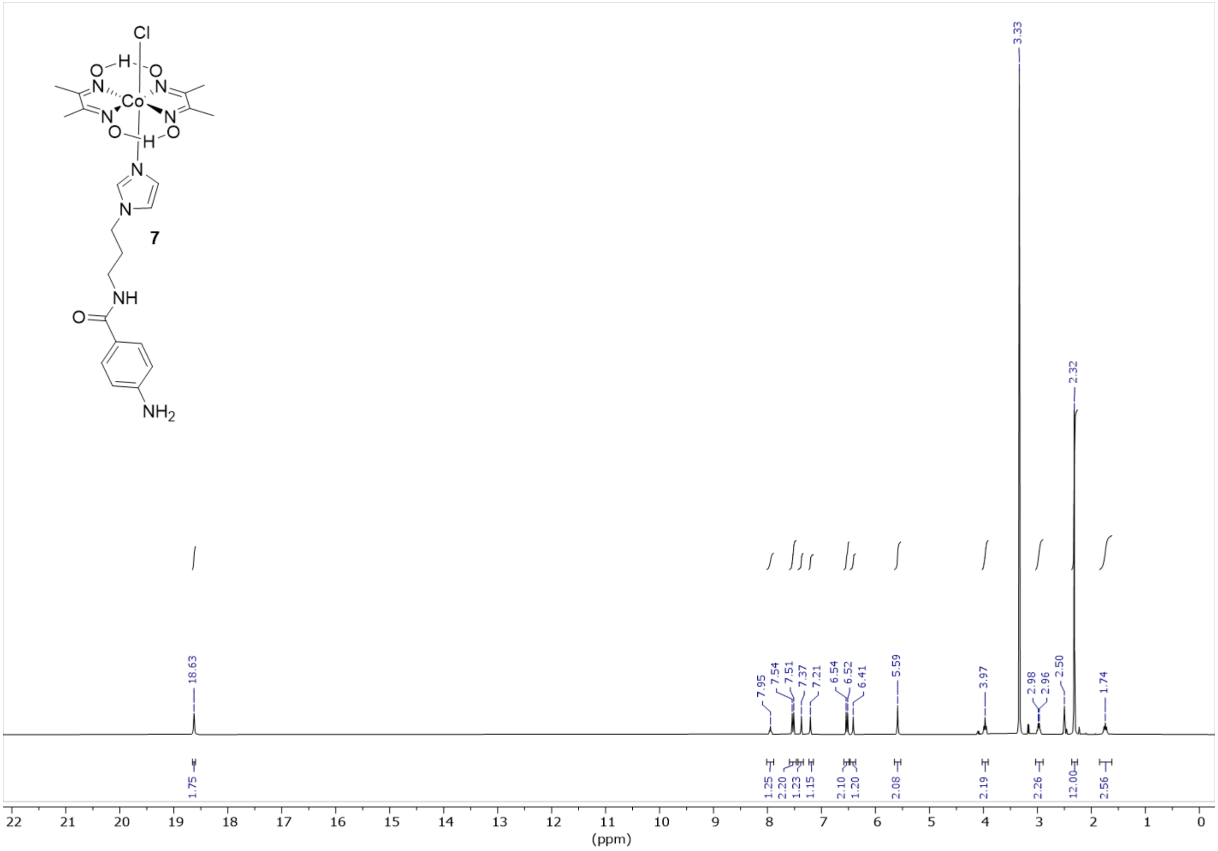


**Figure S9.** $^{1}$H NMR (300 MHz, DMSO-$d_6$) spectrum of compound **7**.

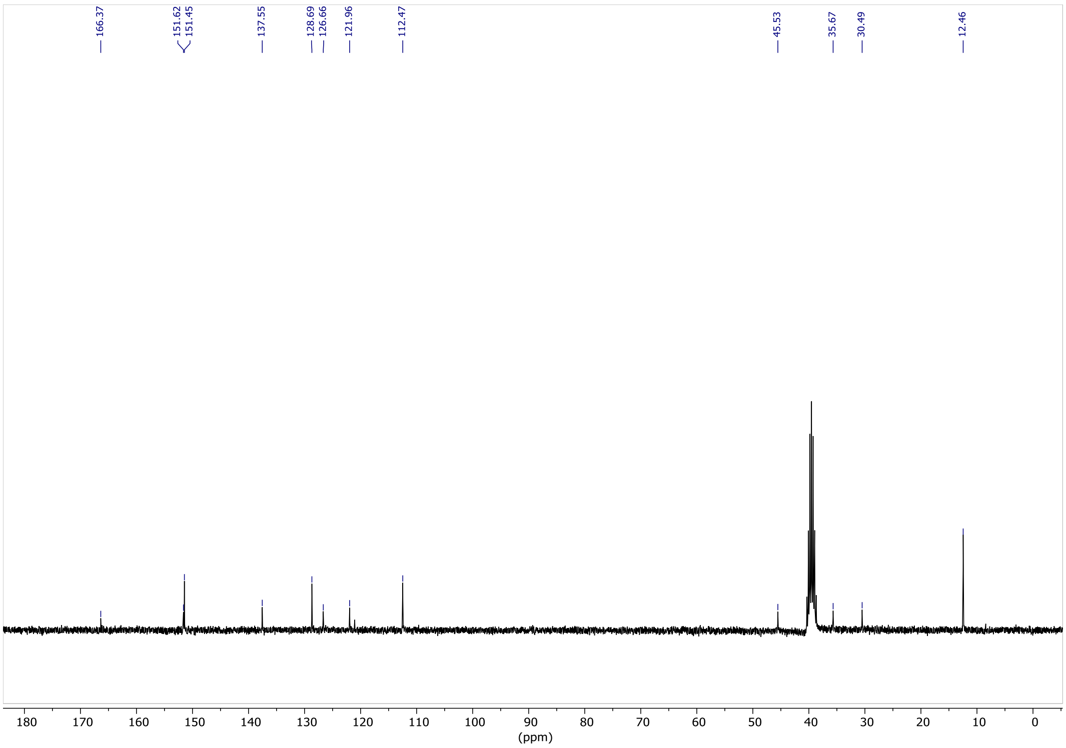


**Figure S10.** $^{13}C\{1\}$ NMR (75.5 MHz, DMSO-$d_6$) of spectrum compound **7**.
